# Real-Time Measurements of Indoor–Outdoor Exchange of Gaseous and Particulate Atmospheric Pollutants in an Urban Area

**DOI:** 10.3390/ijerph20196823

**Published:** 2023-09-25

**Authors:** Elisabeth Alonso-Blanco, Francisco Javier Gómez-Moreno, Elías Díaz-Ramiro, Javier Fernández, Esther Coz, Carlos Yagüe, Carlos Román-Cascón, Adolfo Narros, Rafael Borge, Begoña Artíñano

**Affiliations:** 1 Department of Environment, CIEMAT (Centro de Investigaciones Energéticas, Medioambientales y Tecnológicas), 28040 Madrid, Spain; fj.gomez@ciemat.es (F.J.G.-M.); elias.diaz@ciemat.es (E.D.-R.); javier.fernandezg@ciemat.es (J.F.); esther.coz@ciemat.es (E.C.); b.artinano@ciemat.es (B.A.); 2 Department of Earth Physics and Astrophysics, Complutense University of Madrid, 28040 Madrid, Spain; carlos@ucm.es; 3 Department of Applied Physics, Marine and Environmental Sciences Faculty, INMAR, CEIMAR, University of Cadiz, 11519 Puerto Real, Cádiz, Spain; carlosromancascon@ucm.es; 4 Department of Chemical and Environmental Engineering, Technical University of Madrid (UPM), 28006 Madrid, Spain; adolfo.narros@upm.es (A.N.); rafael.borge@upm.es (R.B.)

**Keywords:** urban air pollutants, real-time I/O measurements, indoor air quality (IAQ), indoor activities, gaseous and particulate pollutants

## Abstract

Air pollution is one of the greatest environmental risks to health, causing millions of deaths and deleterious health effects worldwide, especially in urban areas where citizens are exposed to high ambient levels of pollutants, also influencing indoor air quality (IAQ). Many sources of indoor air are fairly obvious and well known, but the contribution of outside sources to indoor air still leads to significant uncertainties, in particular the influence that environmental variables have on outdoor/indoor pollutant exchange mechanisms. This is a critical aspect to consider in IAQ studies. In this respect, an experimental study was performed at a public site such as a university classroom during a non-academic period in Madrid city. This includes two field campaigns, in summer (2021) and winter (2020), where instruments for measuring gases and particle air pollutants simultaneously measured outdoor and indoor real-time concentrations. This study aimed to investigate the dynamic variations in the indoor/outdoor (I/O) ratios in terms of ambient outdoor conditions (meteorology, turbulence and air quality) and indoor features (human presence or natural ventilation). The results show that the I/O ratio is pollutant-dependent. In this sense, the infiltration capacity is higher for gaseous compounds, and in the case of particles, it depends on the particle size, with a higher infiltration capacity for smaller particles (<PM_2.5_). Additionally, under specific situations of high atmospheric stability, the concentrations of gases derived from traffic tend to accumulate in the room, keeping the background concentrations. These concentrations decreased when room ventilation was produced simultaneously with well-ventilated (high wind speed) external conditions. This suggests that the meteorology and turbulence parameters played a key role in influencing indoor ambient pollution conditions by enhancing the dispersion or accumulation of pollutants. The obtained results highlight the high number of variables involved in the outdoor/indoor exchange of air pollutants and, consequently, how complex its study is. Thus, the knowledge of these factors is critical for understanding the behavior of indoor pollutants and controlling human exposure in indoor environments.

## 1. Introduction

Urban air quality represents a global environmental problem. More than 80% of the urban population is exposed to air pollution levels exceeding the 2006 guidelines of the World Health Organization [1], and a significant majority of the global population (99%) breathes air containing levels of pollutants exceeding the most recent WHO guideline limits [2,3]. Thus, air pollution is the cause of severe respiratory or cardiovascular problems of citizens and is associated with 7 million deaths worldwide annually [4]. Besides health impacts, this issue causes other economic and well-being issues (e.g., life satisfaction, mood, perceived stress or self-esteem) for the population [5]. Thus, air pollution represents a current and future challenge for all governments. In the last decade, political agreements for international cooperation to combat air pollution have been carried out in the United Nations Framework, e.g., the Convention on Climate Change (COP) [6,7]. In Europe, a batch of measures has been launched to address this problem, mostly focused on air pollutant emission abatement. These have been focused on different emission sectors such as road traffic (lowering speed limits, low-emission zones, cleaner public transport, promoting cycling and walking…), residential heating (the insulation of buildings, using cleaner fuels…) or industry (controlling emissions from nonroad mobile machinery such as construction machinery) [8]. Significant efforts have been made at different sectors such as companies, public and private institutions and governmental and local administrative authorities, leading to a successful reduction in ambient concentrations of several pollutants like sulfur dioxide (SO_2_), carbon monoxide (CO) or nitrogen oxides (NO_x_). Nevertheless, air pollution continues to have significant impacts on the European population, particularly in the largest urban and metropolitan areas where the majority of the population lives. According to recent reports [9], the most serious pollutants in terms of harm to human health are particulate matter (PM), NO_2_ and ground-level O_3_. Out of these, and despite the fact that medium levels of tropospheric ozone have increased in urban background areas in the last years, PM and NO_2_ represent the most acute problems in terms of ambient air pollution in European urban areas.

More recently, an important concern for indoor air quality has emerged with the continual improvement in our quality of life. The role of indoor pollution in human exposure has caught scholars’ attention in the last few years, as 4 million premature deaths annually worldwide are related to cooking and heating causes [10]. This problem seems to be more critical in some developing countries exacerbated by the use of unhealthy domestic heating, cooking devices and fuels. Indoor sources have been identified for some air pollutants such as particulate matter, volatile organic compounds (VOCs) or ammonia [11], although emission factor estimations are scarce and present a high variability. However, there is still in general a lack of information on indoor pollution levels due in part to the absence of measurements and specific standards for a great number of indoor pollutants in many countries. Thus, despite the available literature trying to advance on this topic [12], there is still a high uncertainty regarding indoor air quality and its precise role in population exposure. For this reason, the WHO stated that the recently published guidelines apply to both outdoor and indoor environments globally, covering all settings where people spend time [4]. In this regard, between 85 and 90% of the urban population exposure is linked to indoor microenvironments because time is mostly spent indoors according to time–activity pattern studies [13,14,15]. This reinforces the need for a better characterization of levels and sources as a prior stage for developing future abatement and plans for indoor pollution regulation.

Aside from indoor sources, on many occasions, ambient (outdoor) pollution can represent the main contributor to indoor air pollution for some spaces and species. Mechanisms for outdoor/indoor pollutant exchange are a key point, complex and not well understood, partly due to the great number of variables and uncertainties involved. Emerging studies have linked aspects of building design and building operation to air quality in indoor environments [16,17]. The infiltration and penetration factors in buildings have a continuous and important effect on indoor environments [18], and initially, it depends strongly on building envelope leakage [19] such as openings (doors and windows), minute cracks or inadequate sealing. However, other external factors, like the urban air pollution dynamics with its inherent complexity and high temporal and spatial variability, are also involved. For this reason, [20] concludes that dynamic models, taking into account the temporal variability of some involved variables, should be applied to calculate the penetration efficiency. These models must be validated against real-time measurements that provide simultaneous variations in the involved variables.

There are a significant number of experimental studies carried out in indoor environments (schools [11,21,22,23,24], offices [23], residences [25,26], commercial buildings [27] or public transport [28]) that confirm the wide spectrum of conditions and obtained results. Indeed, the complexity of performing indoor measurements is restricted to specific campaigns and short measurement periods, leading to similar studies that are mostly limited. Some recent publications present results on the dynamic variations in indoor/outdoor (I/O) ratio PM levels in schools and homes for a long-term study period, using advanced methods [29]. In this respect, some studies are based on diffusive sampling tubes, providing integrated measurements. This type of sampling does not allow one to study the effect of short-time variations in many involved variables [30], considering an infiltration factor or an indoor/outdoor (I/O) ratio as a fixed parameter. Other studies present real-time measurements for different atmospheric pollutants, mostly based on low-cost sensors, demonstrating the daily variability of the I/O ratio and the importance of considering its dynamic nature [31]. Some recent publications reveal phenomena that are hardly observable with time-averaged techniques. In this context, there is a significant number of variables and features involved in the outdoor/indoor exchange of air pollutants such as the source emission rate, pollutant nature, ventilation mode, indoor activities or atmospheric conditions, and it is necessary to parameterize them over time. One of the most complex aspects are those related to outdoor conditions, especially those that produce very quick changes like meteorology, as their role in the short-term outdoor/indoor exchange is not sufficiently clear. Thus, the study of these factors is critical in the assessment of indoor air quality and human exposure studies.

In this framework, in the course of the AIRTEC-CM project (https://airtec-cm.es/, last access on 3 July 2023), specific experimental campaigns were designed to investigate the interactions between urban air pollutants and meteorology at the local and microscale, focused on the exchange mechanisms between ambient (outdoor) and indoor air pollutants to advance in the comprehensive evaluation of air pollution impacts in cities and population exposure. To this aim, different public locations (a university and hospital building) were selected for the experiments, and two field campaigns, in summer and winter conditions, were carried out in each location. The main objective was to assess the influence of ambient external conditions and some anthropogenic activities on indoor air quality as well as to find out the outdoor/indoor exchange mechanisms for some gaseous and particulate pollutants. In the field campaigns of the hospital building, simultaneous I/O measurements were completed with Black Carbon (BC) and meteorological information obtained by a drone in the vertical profile. Thus, these campaigns will be dealt with in a separate paper addressing a detailed analysis of the I/O values, including the BC vertical distribution. For the sake of clarity and to avoid dwelling on details of different locations and campaigns, we only present the results for the university building, located in an area that usually experiences air quality problems.

Simultaneous measurements were performed in ambient (outdoor) and indoor air in a third-floor university classroom in two seasons (winter 2020 and summer 2021). A large set of instruments were deployed at the site to measure the air concentrations of gaseous pollutants (NO_x_ and O_3_) and several particulate matter parameters in real-time, considering mass concentration at specific size fractions, the number of particles in the ultrafine range or specific elements of its chemical composition (i.e., PM_10_, PM_2.5_, PM_1_, Ultrafine Particle Number Concentration (PNC) and equivalent BC (eBC)). In situ meteorology and turbulence parameters were also monitored to assess the atmospheric dynamics processes for the interpretation of the observed variations in the I/O measurements during the campaigns and their influence during the different experiments.

The general behavior of outdoor and indoor pollutants and their relationships are described, interpreted and discussed in this paper. Conclusions are obtained in light of the atmospheric conditions, external atmospheric pollution and indoor activities influence.

## 2. Methodology

### 2.1. Area of Study and Experimental Site

Madrid, with 3.3 million inhabitants (2021), is the most populated city in Spain. With a car fleet of around 4.5 million vehicles, the city occasionally experiences air pollution problems that can be mostly associated with traffic emissions. This source and heating device emissions during the cold season are the main pollutant sources of this city, as there are no significant industrial sources nor other emission sources influencing the urban area.

In the last ten years, the air quality in Madrid experienced a clear improvement in general terms, and several air pollutants (SO_2_, CO, PM_10_, PM_2.5_ and NO_2_) exhibit a decreasing trend that seems to continue according to the latest reports [32,33,34]. This fact can be attributed, among other factors, to the different Air Quality Plans, technological improvements in the main emission source, road traffic and changes in heating devices and fuels [35,36,37]. Other pollutants like benzene do not share this downward trend, whereas others like ozone reveal an opposite and increasing trend that has been related to changes in the NO_2_/NO ratio associated with variations in the emission factors and the vehicle fleet composition of the Madrid region and, in general, in Spain [37].

Despite these partial improvements, the EU mean annual limit value for the health protection of NO_2_ (Directive 2008/50/CE: 40 µg m^−3^, [38]) has still been exceeded in the last years 2018, 2019, 2020 and 2021, and under specific atmospheric conditions, mostly in fall and winter, the NO_2_ hourly limit value (200 μg m^−3^) is also exceeded at any station of the municipal monitoring network. In these specific adverse atmospheric situations, the urban area experiences severe pollution episodes, lasting from several days to more than one week, which requires the implementation of short-term action plans (i.e., NO_2_ protocol) to diminish the ambient concentrations and prevent adverse effects on the urban population [39]. Particulate matter (PM_10_ and PM_2.5_) has never exceeded the legislation limits in the last five years, although annual mean concentrations are clearly above the WHO guidelines of 2021 (45 and 15 µg m^−3^, respectively) [4].

**Figure 1:**
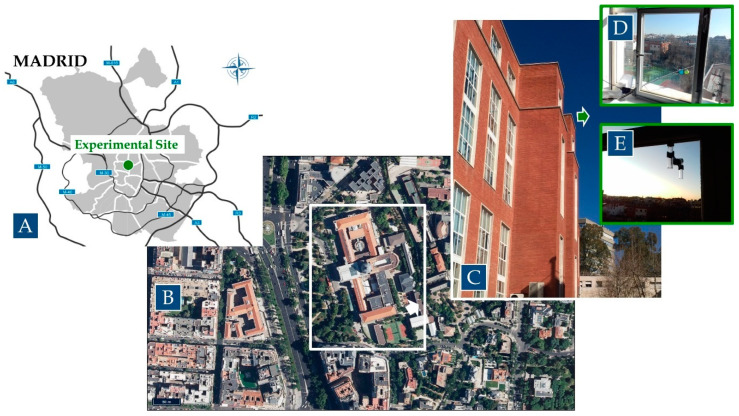
Location of the experimental site in Madrid city (**A**). Surroundings of the site, with the university’s main building inside the white rectangle (**B**), and the outside of the classroom in the university building (**C**). (**D**,**E**) are pictures of the sampling inlets for outdoor ambient air measurements and the passive tubes, respectively.

The experimental site (university building) is located on a main avenue (Paseo de la Castellana) of the Madrid city center (Figure 1), so the influence of traffic emissions is significant as revealed by the measurements of the air quality (AQ) monitoring station (#Castellana station) of the municipal network. This station has recorded, during the last four years (2018–2021), NO_2_ annual means of 39, 34, 28 and 29 µg m^−3^, respectively, showing the general decline observed in many other stations and urban areas in Europe associated with the emission changes induced by the COVID19 pandemic. It must be noticed that, on average, NO_2_ pollution levels in Madrid in 2021 were 26% lower than the average value recorded in the 2010–2019 period, whereas in 2020, the reduction was 31% [40]. While the experimental winter campaign (2020) was carried out during the prelockdown period, the summer (2021) campaign was carried out postlockdown. Thus, the pollution situation should not be affected by lockdowns. This fact will be discussed below.

### 2.2. Experimental Deployment and Instrumentation

For this study, experimental campaigns were carried out on 6 February to 1 March 2020 and 14–23 June 2021, representative of winter and summer conditions, respectively.

The ambient air pollution was characterized at the street level by the #Castellana station of the municipal network. This station provided NO and NO_2_ measurements at the street level as well as particulate matter (PM) in the PM_10_ and PM_2.5_ size fractions with a frequency of 10 min. These measurements were complemented for this study with some additional instrumentation like an Aethalometer for obtaining eBC (equivalent Black Carbon) mass concentrations and instruments devoted to estimating turbulence parameters from high-frequency atmospheric measurements. Additionally, a meteorological mast was installed at the building roof (22 m above ground level (AGL)) to provide nonperturbed measurements of standard meteorological parameters. A detailed description of the meteorological information can be found below.

Instruments for the outdoor/indoor air quality study were installed inside a third-floor university classroom (~15 m AGL) in the south–southwestern corner of the university building. All the instruments were operating inside the room, whereas an inlet was installed for external air sampling through a window of the same room. This window was sealed after the installation of the sampling line to obtain representative indoor conditions of the closed windows.

Periods of different anthropogenic activities performed inside the room (instrumentation maintaining, sampling or data backup) which indicated human presence and periods of natural venting (window opening) were identified and recorded for the interpretation of the observed variations in the I/O measurements during the campaigns.

#### 2.2.1. Air Pollutant Measurements

Table S1 (in the Supplementary Materials) shows the main details of the instruments, parameters and type of measurement (indoor/outdoor) performed during the campaigns.

Outdoor and indoor gaseous (NO, NO_2_ and O_3_) concentrations were measured with standard analyzers previously intercompared and calibrated in the laboratory. Two analyzers for each parameter (NO_x_ and also O_3_ in the 2021 campaign) were used for measurements inside and outside the room. With this configuration, uncertainties derived from different monitoring systems or bias due to different ambient conditions (humidity and temperature) [41] are avoided. Sampling inlets were designed for outdoor measurement by passing the sampling Teflon^©^ tubes through a perfectly sealed window to prevent the air mass exchange with closed windows.

Palmes-type passive diffusion tubes (PDT) were deployed outdoors and indoors at the measurement site during each campaign. Weekly variations in the NO_2_ average concentration levels were assessed through two weekly periods with a solution of 20% TEA/water [42,43]. Four tubes were displayed at the AQ station in each weekly measurement period.

Particulate matter mass concentrations at different size ranges (PM_10_, PM_2.5_ and PM_1_) and Particle Number Concentrations (PNCs) in the ultrafine range were measured outdoors and indoors in the experimental room during the study. The eBC concentrations, considered a good tracer of the traffic-origin combustion emissions, were also measured outdoors and indoors.

The I/O ratios were calculated from the database obtained for each field campaign. According to the lognormal distribution property of ratio indicators, the geometric mean is the main statistical parameter used to present data. Thus, the geometric mean of hourly ratios was determined. I/O ratios < 1.0 indicated that contributions from indoor sources are less than those from outdoor ones, while I/O ratios > 1 showed the dominance of indoor sources. In this study, the 95% confidence intervals for the I/O ratios were also calculated. When value 1 is within the 95% confidence interval for the geometric mean, it cannot be concluded if the highest pollutant concentration is in an indoor or outdoor environment.

#### 2.2.2. Meteorology and Turbulence Parameters

Standard meteorological variables: wind speed (WS) and direction (WD), air temperature (T), relative humidity (RH), atmospheric pressure and global (incoming) solar radiation were measured at a meteorological station located on the roof of the building. Micrometeorological parameters were obtained at street level (2 m AGL) by a sonic anemometer (Campbell Scientific, Logan, UT, USA) to study the influence of the atmospheric stability and turbulent mechanisms for pollutant diffusion and its influence on the outdoor/indoor exchange. These parameters (Turbulent Kinetic Energy (TKE), friction velocity (u*) and Sensible Heat flux (SH)) were calculated from these data, considering 10 min averages for variance and covariance evaluations.

## 3. Results and Discussion

### 3.1. General Overview

Figures S2 and S3 show the indoor/outdoor time series obtained in the university building during the sampling period during the winter (2020) and summer (2021) campaigns, respectively.

The influence of the ambient conditions (meteorology and air quality) on the outdoor/indoor exchange of air pollutants is a key aspect considered in this study. Meteorology can modulate the thermodynamic state of the atmosphere, turbulence and stability and therefore the dispersion conditions that act on the ambient pollutant concentrations, which in turn influence the indoor concentrations.

The meteorological situation during the experimental campaigns was characterized, on a large/synoptic scale, by the prevalence of high-pressure systems that favored the atmospheric stability during most parts of the measuring campaigns. In relation to the normal situation in Madrid, provided by a climatological study of anomalies pressure at surface and Z500 levels, the atmospheric conditions were more stable during winter 2020 and more unstable during summer 2021. This played a key role in the ambient concentrations of some pollutants as will be analyzed below.

**Figure 2:**
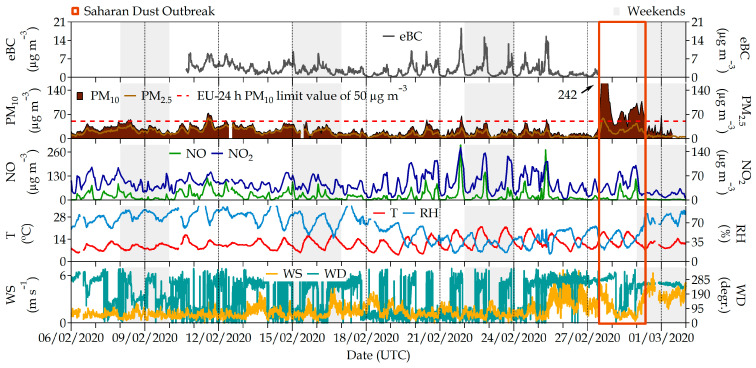
Air pollutants PM_10_, PM_2.5_, NO, NO_2_ and eBC recorded at the #Castellana station during the field campaign in February 2020. The two lower panels correspond to meteorological parameters (temperature (T), relative humidity (RH), wind speed (WS) and wind direction (WD)) recorded at the station located on the ETSII building roof.

**Figure 3:**
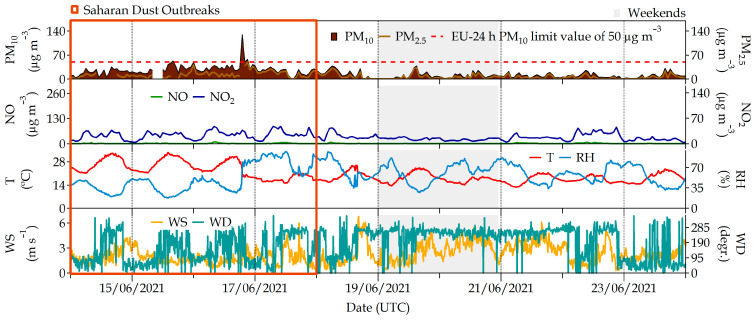
Air pollutants PM_10_, PM_2.5_, NO, NO_2_ and eBC recorded at the #Castellana station during the field campaign in June 2021. The two lower panels correspond to meteorological parameters (temperature (T), relative humidity (RH), wind speed (WS) and wind direction (WD)) recorded at the station located at the ETSII building roof.

In winter (February 2020), a high-pressure system covered a great part of the Iberian Peninsula, including the center and the Madrid area. Low wind speeds and surface temperature inversions characterized the atmospheric situation during this period, inhibiting ventilation and enhancing pollutant accumulation. The most critical days of the episode were 19–25 February when the highest values of gaseous pollutants and eBC concentrations were recorded. During this campaign, NO and NO_2_ at the AQ station reached hourly maxima of 300 and 152 µg m^−3^, respectively, which was rather higher than the summer campaign maxima (15 and 44 µg m^−3^, respectively) (Figure 2 and Figure 3). In the summer, reductions in NO_x_ concentrations are the result of an increase in ozone titration.

Only on several days (13, 16–17, 25–27 February) did a light atmospheric instability caused by a relative low-pressure system approaching the Iberian Peninsula perturb these high-stability conditions. As a consequence, the wind speed and turbulence parameters experienced moderate increments during these days, and occasional wind gusts and small precipitation volumes were eventually recorded, producing a decrease in air pollutant concentrations.

Another interesting event during the winter field campaign was observed on 27 February when an intense Saharan dust outbreak reached the Madrid area, associated with a noticeable increase in PM_10_ concentrations that reached an hourly maximum of 242 µg m^−3^ (Figure 2). These events are typically produced by long-range transport processes of Saharan dust. They can be clearly identified, and their impacts on the surface PM concentrations are documented following a well-established and validated methodology [44,45]. The consequence of this dust outbreak was an increase in ambient PM concentrations, mainly the coarsest fraction PM_10_, but also to a minor extent the smaller size fractions (PM_2.5_ and PM_1_).

**Figure 4:**
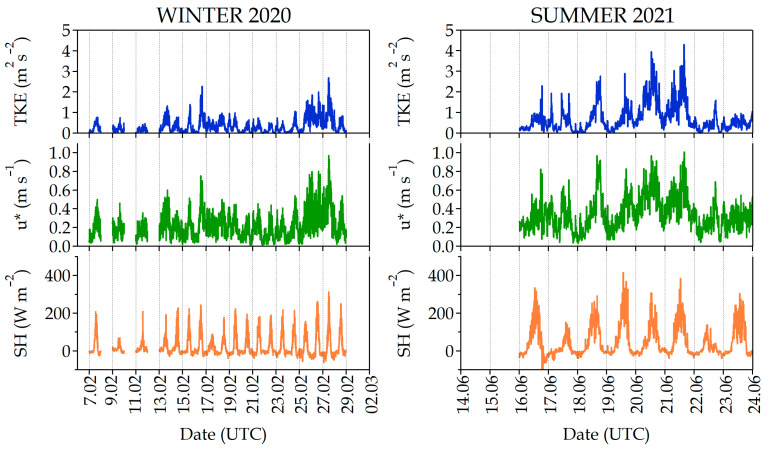
Turbulence parameters (Turbulent Kinetic energy (TKE), friction velocity (u*) and Sensible Heat flow (SH)) recorded at the #Castellana station during the field campaigns in February 2020 (winter 2020) and June 2021 (summer 2021).

During the summer (14–23 June 2021) campaign, most days were under the influence of the Azores anticyclone, and fair weather with high solar radiation and moderate winds presented the general picture of the meteorological situation. This situation favored ground heating and thermal convection, which enhanced dispersion conditions, as can be observed in the turbulence parameters shown in Figure 4. These fair-weather conditions also favored the development of local circulations in the summer during the whole campaign, although there was also an event of atmospheric instability and precipitation (17 and 22 June 2021) according to the data provided by the Spanish national meteorological office (AEMET, https://www.aemet.es/, last access on 3 July 2023). Ambient pollutant concentrations were in general lower during the summer campaign than in the winter one, as meteorological conditions favored the ventilation and dispersion conditions during this experimental period (Figure 2 and Figure 3). In addition, the mixing layer height was, as expected, higher than in the winter. Only O_3_, a photochemical pollutant whose formation is enhanced by solar radiation, reached its maximum value in the summer.

Turbulence parameters, such as the Turbulent Kinetic energy (TKE), friction velocity (u*) and Sensible Heat flow (SH) measured at the street level can be used as a proxy of the atmospheric dispersive conditions. Figure 4 shows the extremely different conditions in the winter and summer, with clearly lower values of the three turbulence parameters (TKE, u* and SH) in February 2020, indicating a very stable atmosphere (except during the above-mentioned instability periods, especially 26–27 February) as opposed to the summer campaign when higher values and a marked daily pattern of these parameters can be observed, whereby it is very obvious that the SH is associated with the sun heating cycle for most days.

Local winds at the measurement site exhibited a wind rose distribution in the winter campaign with two main wind directions around the W and NE sectors (Figure S1). This last sector mainly corresponded to the nocturnal period, as can be seen from the daytime and nighttime distributions. The summer wind roses showed these same dominant directions, with higher wind speeds in general and an NE direction component as more intense winds came from this sector. These dominant directions during night and day are impacted by the local/regional thermally driven flows that typically develop in the region when stable conditions (weak synoptic forcing) dominate.

### 3.2. Mean Indoor/Outdoor Ratios

Table 1 shows the mean I/O ratios for the different pollutants obtained from the continuous measurements of monitors and also from the NO_2_ concentrations obtained with passive tubes. The I/O pollutant concentrations (mean and range (min–max)) for each field campaign are shown in Table S2.

For particulate matter, the lowest I/O ratios were obtained for PM_10_, varying from 0.02 to 1.07 with a seasonal mean of 0.13 and 0.28 in the winter and summer, respectively. The PM_2.5_ I/O ratios were from 0.05 to 0.87 with mean values of 0.18 and 0.37 in these seasons, and the PM_1_ I/O ratio varied from 0.31 to 1.25 (mean of 0.54) in the summer campaign. The minimum I/O ratios of PM_x_ corresponded to periods of nonoccupation and maximum ones to periods of occupation. Traffic-related pollutants such as eBC, which is mostly found in the finest fraction, presented I/O ratios between 0.18 and 2.10 and between 0.42 and 2.55 in the winter and summer, respectively, whereas the I/O ratio varied from 0.11 to 1.17 and from 0.37 to 1.47 for ultrafine particles in these seasons. In the case of these pollutants, minimum and maximum I/O ratios were observed during periods of nonoccupation. Different studies performed in urban schools like [24] and those compiled by [21] report I/O ratios for any of these parameters under a wide range of conditions and locations in the range of 0.60 to 0.92 for eBC, which is within the range observed in this study, and a minor variation of 0.80 to 1.27 for PM_2.5_ or PM_10_, in which [3] found large variations in Bangladesh’s hospitals (from 0.72 to 1.50). Only a few works in the literature report on Ultrafine Particle Number Concentration (PNC) ratios; for example, [21] found a ratio of 0.66 obtained during school hours in the framework of the BREATHE project that studied 39 schools in Barcelona. This value is within the range obtained in the present study that shows, in general, a variation in I/O ratios with the particle size; i.e., the higher the size, the lower the I/O ratio. Atmospheric conditions also influence these ratios, and summer conditions seem to enhance the infiltration capacity of particles due to the enhanced turbulence favored by the convective conditions, among other factors.

Concerning gaseous pollutants, the lowest I/O ratios in this study were recorded for O_3_, although these data corresponded only to the summer campaign. In the winter, low O_3_ concentrations are expected due to the photochemical nature of this pollutant and the NO titration reaction that destroys ozone. This is particularly evident in the case of traffic sites and NO accumulation situations like the winter episode of this study (Table 1). As an example, O_3_ concentrations measured during the winter and summer campaign periods at a near urban traffic station of the network (#Escuelas Aguirre, ~2 km south–southeast of the sampling site) were 23.5 and 59.5 μg m^−3^, respectively. Unfortunately, the #Castellana station does not provide O_3_ measurements, so the observations from the nearby and similar #Escuelas Aguirre station were used for outdoor analyses in the winter.

NO and NO_2_ exhibited a particular behavior with I/O ratios ranging from 0.34 (for NO in winter) to values > 10 (for both parameters), with mean values in both periods >1.0 (Table 1). This means that pollutants were accumulated indoors. The I/O NO_2_ ratios for passive tubes were ~1, which is particularly significant in this study given that the I/O ratios were obtained with monitors ranging between 0.77 and 1.59. This phenomenon has also been observed in other studies [31,46], and to be understood, it is necessary to see the temporal evolution with a higher resolution than we have.

There is great variability in I/O ratios for NO and NO_2_ found by other authors in different locations or buildings such as hospitals [3], schools and homes [21,46,47] or apartments [31]. This variability, for instance, in the NO_2_ I/O ratio that goes from 0.63 in Barcelona [21] to 1.16 in Antwerp [46] highlights the wide range of concentrations that the population is exposed to and the complexity of the air quality assessment in indoor environments.

This complexity is evident for pollutants from an anthropogenic external source and is considered to exclusively have a traffic origin, like NO or NO_2_. Nevertheless, it could be increased for PM or VOCs whose sources can be found both in indoor and outdoor environments [11].

### 3.3. Real-Time Indoor/Outdoor Ratio Variations

Real-time measurements provide an interesting input for analyzing the time variations in indoor concentrations that are influenced by internal or external contribution variations, reinforcing the proposal for using a dynamic I/O ratio for exposure assessments [31]. These variations were also observed in this study; in this case, they are associated with outdoor concentrations, which depend on the meteorological conditions, but also on the activity (occupancy, open windows or both) within the room, as we will see next.

**Figure 5:**
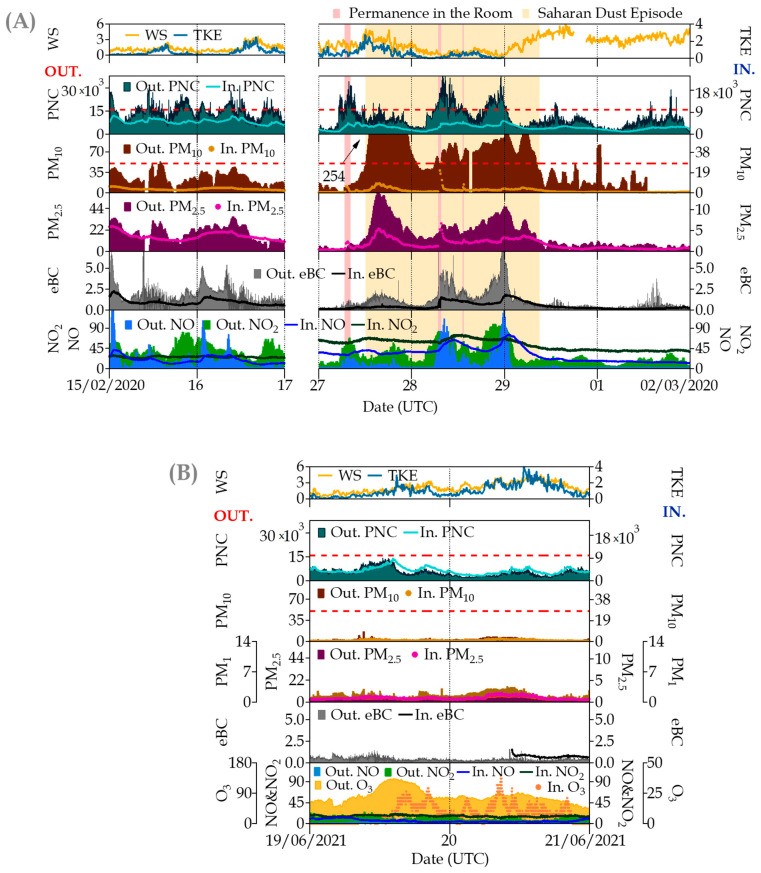
Simultaneous time series of indoor/outdoor (I/O) pollutants measured at the university site in some non-occupancy periods (weekends) during (**A**) winter and (**B**) summer campaigns. Parameters (units) are denoted in the graphs as follows: WS = wind speed (m s^−1^); TKE = Turbulent Kinetic Energy (m^2^ s^−2^); PNC = Ultrafine Particle Number Concentration (cm^−3^); PM_10_, PM_2.5_ and PM_1_ = particulate matter mass fractions (μg·m^−3^); eBC = equivalent Black Carbon (μg·m^−3^); and NO, NO_2_ and O_3_ = Trace gas pollutants (all in μg·m^−3^). The red dotted line in PNC represents the optimal detection range of the indoor CPC (0–1 × 10^4^ cm^−3^); in PM_10_, this dotted line represents the European 24 h standard of 50 μg m^−3^ for outdoor ambient air levels. In the graph, the prefix “Out.” refers to outdoor measurements, whereas “In.” indicates indoor measurements. Note: in order to show the influence of the Saharan intrusion on indoor measurements during non-occupancy periods, permanence in the room was incorporated into the graph.

Figure 5 shows the results of both campaigns during some non-occupancy periods. In the absence of known indoor sources in the university building, indoor concentrations experience a clear influence from outdoor ones. A similar trend can be observed for some pollutants with a clear origin in traffic in Madrid [2,48] like PNC, PM_2.5_ and eBC, whereas for others like PM_10_, from other sources, the response of indoor concentrations is not so significant, even for the case of the intense Saharan dust intrusion that hardly influenced the indoor concentrations as [49] identified during dust storm events in residential homes in Kuwait. In that figure, the same scales were used for winter (panel A) and summer (panel B), evidencing the great difference in ambient concentrations among the winter and summer campaigns, much lower for the latter.

In the case of other gaseous pollutants like NO or NO_2_, this behavior cannot be recognized as there are chemical mechanisms involved in the accumulation and reaction of these compounds that must be investigated separately. While under several circumstances indoor NO evolves accordingly to outdoor increases, it seems that this gas, with low reactivity, can be accumulated indoors, keeping an indoor background level that can be even greater than outdoor values under conditions (29 February and 1 March) of high wind speed (>3 m s^−1^). In this regard, [17] did not find a correlation with building permeability for I/O NO ratios (from 0.5 to 1). The NO accumulated in the room only decreases with ventilated conditions favored by a wind gust that cleans the atmosphere and also causes indoor pollutants to be dispersed, reinforcing the role of meteorology on indoor pollution.

A delay (up to several hours) was observed to reach the indoor concentrations at their maximum levels with respect to the external ones. This delay was also observed and quantified by [50] as ~1 h for eBC while other studies [51,52,53] identified delay times from one to a few hours for the Ultrafine Particle Number Concentration. In this work, similar delay times were found, although there is in general a great variability among the species and the different experimental conditions.

**Figure 6:**
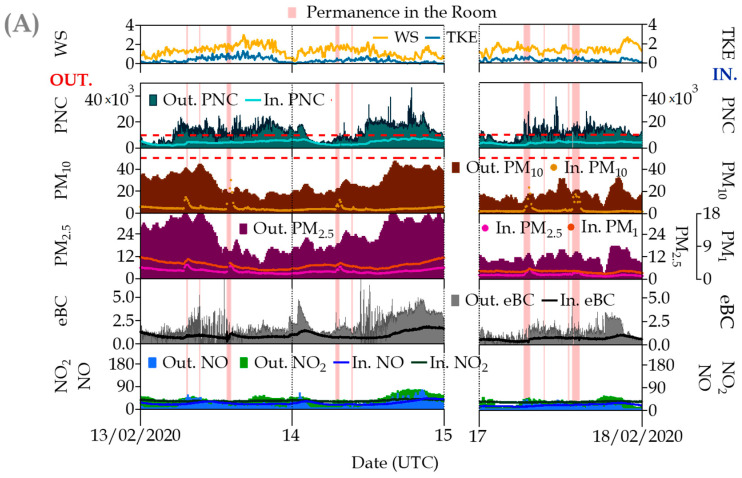

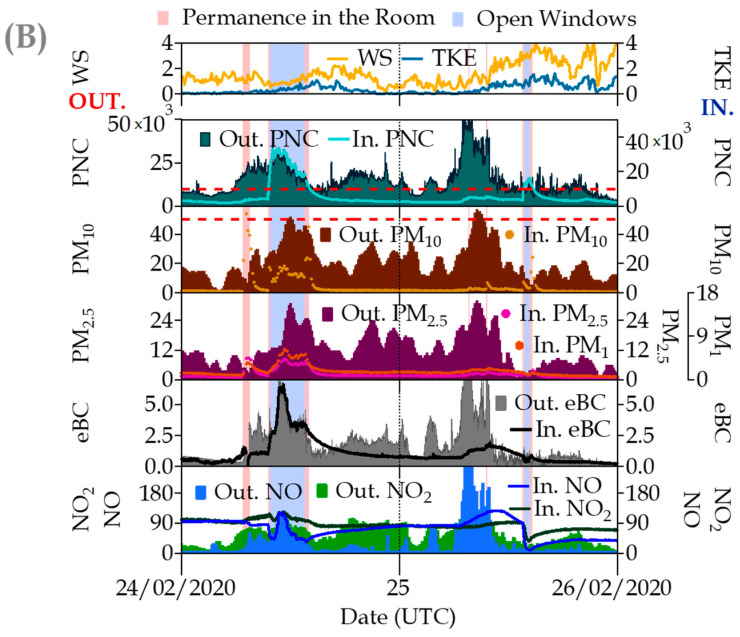
Simultaneous time series of indoor/outdoor (I/O) pollutants measured at the university site in some occupancy periods during the winter campaign: (**A**) Permanence in the room and (**B**) Permanence in the room + open windows. Parameters (units) are denoted in the graphs as follows: WS = wind speed (m s^−1^); TKE = Turbulent Kinetic Energy (m^2^ s^−2^); PNC = Ultrafine Particle Number Concentration (cm^−3^); PM_10_, PM_2.5_ and PM_1_ = particulate matter mass fractions (μg·m^−3^); eBC = equivalent Black Carbon (μg·m^−3^); and NO, NO_2_ and O_3_ = Trace gas pollutants (all in μg·m^−3^). The red dotted line in PNC represents the optimal detection range of the indoor CPC (0–1 × 10^4^ cm^−3^); for PM_10_, this dotted line represents the European 24 h standard of 50 μg m^−3^ for outdoor ambient air levels. In the graph, the prefix “Out.“ refers to outdoor measurements, whereas “In.“ indicates indoor measurements.

Perturbations of indoor pollutant concentrations associated with human presence or room occupation were observed in this study. As has been evidenced in other studies [11,24], there are indoor sources (resuspension, clothes, chalk or building/furniture material deterioration) related to human presence and determined anthropogenic activities, which can interfere with observed concentrations and therefore the I/O ratios of some pollutants during specific periods. In Figure 6A, periods of human presence in the room for instrument maintenance are indicated for the winter campaign. An increase in particulate matter concentrations from the resuspension of soil particles was associated with these maintenance periods, affecting the three size fractions PM_10_, PM_2.5_ and PM_1_, mostly the coarsest fraction (e.g., on 24 February ~07:00 UTC indoor and outdoor PM_10_ values were 54 and 7 µg m^−3^, respectively), whereas no significant variations were observed for the ultrafine particles (PNC) due to these anthropogenic activities.

However, a clear perturbation was documented during those periods of active room ventilation by window opening (Figure 6B). For the 24 February event, all pollutant concentrations inside the room showed, without any exception, a fast increase (~30 min), especially PNC and eBC, which are the best tracers of traffic emissions, and they could reach the external ambient levels (~2.3 × 10^−4^ particles cm^−3^ and ~2.7 µg m^−3^, respectively) due to an accumulation situation, with a low wind speed (0.9 ± 0.2 m s^−1^) and turbulence (0.4 ± 0.2 m^2^/s^2^) degree (Figure 4). It is worth mentioning the rather different behavior of the indoor pollutants during the opening window event on 25 February coinciding with a ventilated situation (relatively higher wind speed (3.8 ± 0.5 m s^−1^) and turbulence (0.8 ± 0.2 m^2^/s^2^)) that, except for the ultrafine particles (up 1.5 × 10^−4^ cm^−3^) and PM_10_ (up 7 µg m^−3^), produced a pollutant cleanup of the room despite the shorter duration of the window opening. The pollutant levels in the room gradually returned to the previous values after closing the window, even taking hours before the I/O concentration reached equilibrium.

**Figure 7:**
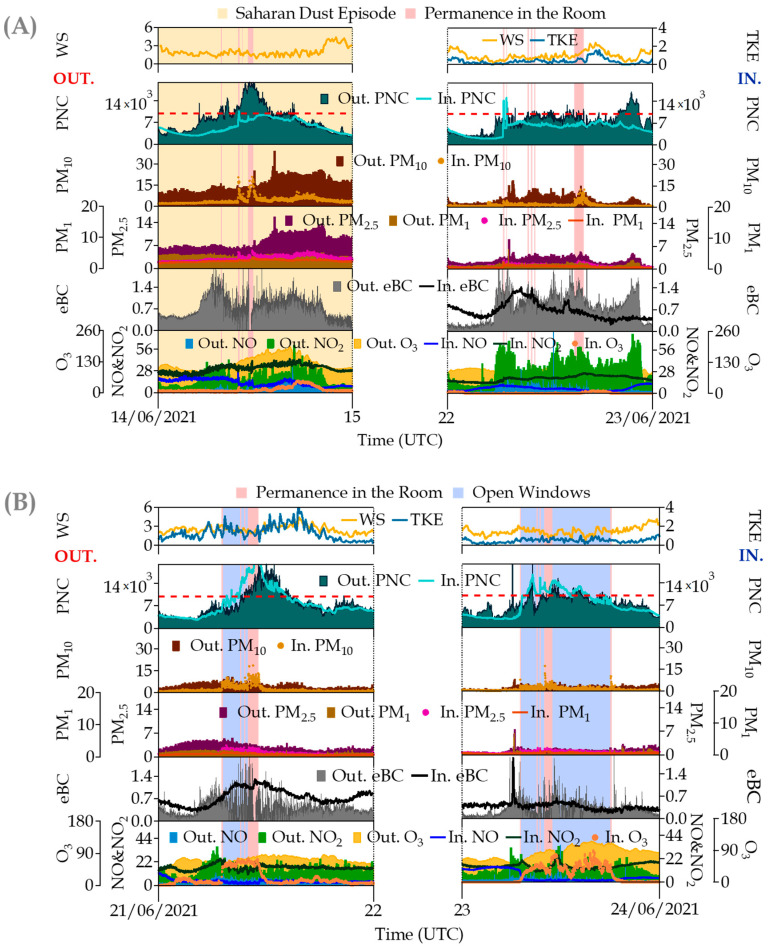
Simultaneous time series of indoor/outdoor (I/O) pollutants measured at the university site in some occupancy periods during the summer campaign: (**A**) Permanence in the room + Saharan dust episode (14 June 2021) and Permanence in the room (22 June 2021) and (**B**) Permanence in the room + open windows. Parameters (units) are denoted in the graphs as follows: WD = wind direction (m s^−1^); TKE = Turbulent Kinetic Energy (m^2^ s^−2^); PNC = Ultrafine Particle Number Concentration (cm^−3^); PM_10_, PM_2.5_ and PM_1_ = particulate matter mass fractions (μg·m^−3^); eBC = equivalent Black Carbon (μg·m^−3^); and NO, NO_2_ and O_3_ = Trace gas pollutants (all in μg·m^−3^). The red dotted line in PNC represents the optimal detection range of the indoor CPC (0–1 × 10^4^ cm^−3^). In the graph, the prefix “Out.“ refers to outdoor measurements whereas “In.“ indicates indoor measurements.

Figure 7, corresponding to the summer campaign, illustrates the effect of occupancy, including a new situation characterized by a Saharan dust outbreak (panel A) and occupancy and open windows (panel B). Again, a small increase in the particulate matter concentrations is observed for PM_10_, PM_2.5_ and PM_1_ during the occupancy periods (e.g., indoor values on 14 June at 11:00 UTC ranging from 2.9 to 20.8, 2.9 to 5.5 and 2.5 to 3.5 for PM_10_, PM_2.5_ and PM_1_, respectively), not affecting the other pollutants. However, the effect of the window opening is clearly observed in all of them despite the lower concentrations in comparison with the winter campaign. In this case, ambient O_3_ entering the room destroys the NO background present indoors whereas NO_2_ increases by titration to reach the outdoor concentrations. A nocturnal accumulation in the room can be observed as well in this last pollutant, with I/O NO_2_ ratios ranging between 0.9 and 1.8.

## 4. Conclusions

Real-time measurements of indoor and outdoor (and meteorology) pollutants were conducted during winter 2020 and summer 2021 in a classroom in a university building located in downtown Madrid. This study aims to evaluate the role of outdoor (pollutants and meteorology) conditions and indoor activities on indoor air quality.

The atmospheric stability situation, given by turbulence parameters, affected the dispersion conditions and therefore the ambient concentration of all the studied pollutants. This evidenced the influence of outdoor pollutants on their indoor levels, although not in the same way for all pollutants and the two seasons.

The gaseous pollutants presented higher I/O ratios than the particulate matter, indicating a greater infiltration capacity. The atmospheric external conditions were relevant as the high outdoor concentrations observed in the winter episodic conditions for NO or NO_2_ made these pollutants accumulate (I/O ratios > 1) within the room via infiltration; i.e., the indoor concentrations were higher than the outdoor ambient concentrations. These conditions remained until the room was ventilated (window opening), although this type of ventilation can also produce the opposite effect, i.e., an increase in indoor NO_x_ concentrations. In the summer, the window opening favored the entrance of O_3_-enriched air that reacted by destroying the NO by titration. Under these conditions, indoor concentrations increased but never reached the outdoor ambient levels.

Particle size is an important feature of the infiltration factor and must be taken into account in its parameterization. The smallest particles in the ultrafine range, more directly related to traffic emissions, as eBC concentrations confirm, had a greater infiltration capacity with the ensuing consequences for population exposure and health-impact assessments. Ultrafine particles should be a candidate parameter for this type of study and air-quality-impact assessment.

Additionally, a seasonal variation was found in the I/O ratios, which exhibit, in general, higher values in the summer conditions. This can be related to the higher turbulence degree of the atmosphere due to the enhanced convection, favoring the infiltration. Oppositely, and despite contributing to the pollutant’s infiltration in the building, the dispersion conditions in the summer reduced the outdoor concentration levels (except for the O_3_). In addition, rapid changes in the outdoor concentrations associated with meteorological conditions can result in high I/O ratios.

As mentioned above, anthropogenic activities, especially those associated with room occupancy (in this case for instrumental maintenance), had a direct impact on the particulate matter concentrations. The coarsest fractions increased during the people’s presence in the room, probably due to particle resuspension as a main indoor source. This showed, on occasion, that the PM_10_ indoor concentration was greater than the outdoor one. However, the clearest effect can be associated with the natural ventilation by window opening, which produced an increase in all pollutant concentrations inside the room, especially the ultrafine particles and their main components, PNC and eBC for episodic stagnant situations; however, a cleaning-up effect occurred when the action took place under high dispersive conditions (i.e., high wind speed and turbulence).

This work contributes to the existing literature by extensively examining the role of local micrometeorology and the importance of the real-time monitoring of meteorological conditions like wind speed and turbulence for abatement measures in indoor environments. A major implication of our findings is that they can result in significantly improved air quality and healthier environments, controlling the influence of the transfer of air pollutants between the outside and inside. Poor indoor air quality harms health. Thus, further studies should be carried out due to the variability of the results and conditions and the number of parameters involved; among them, the building characteristics are not addressed in this study.

## Figures and Tables

**Table 1:** Geometric mean I/O ratios of pollutants (PNC, eBC, PM_10_, PM_2.5_, PM_1_, NO, NO_2_ and O_3_) measured at the university site during winter (6 February to 1 March 2020) and summer (14–23 June 2021) campaigns. I/O ratios for NO_2_ measured by passive diffusion tube are included in the table. The geometric mean of hourly ratios was used to calculate the mean I/O ratio and 95% confidence intervals (in brackets) of the pollutants measured in this study.

	TKE ^(1)^ (m^2^/s^2^)	PNC	PM_10_	PM_2.5_	PM_1_	eBC	NO	NO_2_	NO_2_ Passive Tubes	O_3_
Winter campaign										
All periods	0.34	0.36 [0.34, 0.37]	0.13 [0.12, 0.13]	0.18 [0.17, 0.19]	-	0.61 [0.58, 0.64]	3.22 [2.88, 3.61]	1.19 [1.13, 1.26]		-
Week 1 (10 (11:05 UTC)–17 (13:05 UTC) February)	0.32	0.39 [0.37, 0.41]	0.17 [0.16, 0.18]	0.20 [0.19, 0.21]	-	0.59 [0.56, 0.63]	1.54 [1.36, 1.74]	0.77 [0.73, 0.81]	0.82	-
Week 2 (17 (13:06 UTC)–26 (13:22 UTC) February)	0.31	0.32 [0.29, 0.35]	0.11 [0.10, 0.13]	0.16 [0.15, 0.18]	-	0.62 [0.57, 0.68]	5.01 [4.00, 6.26]	1.71 [1.55, 1.88]	0.89	-
Stagnation episode days (19–25 February)	0.26	0.29 [0.26, 0.33]	0.10 [0.09, 0.12]	0.15 [0.13, 0.17]	-	0.61 [0.55, 0.68]	5.52 [4.24, 7.18]	1.69 [1.53, 1.88]	-	-
Summer campaign										
All periods ^(2)^	0.75	0.79 [0.76, 0.82]	0.28 [0.27, 0.30]	0.37 [0.36, 0.39]	0.54 [0.52, 0.56]	0.94 [0.88, 1.00]	2.56 [2.31, 2.82]	1.25 [1.19, 1.31]		0.06 [0.05, 0.07]
Week 1 (14 (9:56 UTC)– 21 (10:00 UTC) June)	0.77	0.78 [0.74, 0.82]	0.26 [0.24, 0.27]	0.34 [0.33, 0.36]	0.51 [0.49, 0.53]	0.88 [0.83, 0.93]	2.72 [2.43, 3.04]	1.28 [1.21, 1.35]	1.15	0.06 [0.05, 0.07]
Week 2 (21 (10:01 UTC)–23 (11:22 UTC) June)	0.73	0.85 [0.75, 0.89]	0.36 [0.32, 0.41]	0.46 [0.42, 0.50]	0.63 [0.57, 0.70]	1.06 [0.91, 1.23]	1.86 [1.54, 2.25]	1.16 [1.05, 1.27]	1.04 ^(3)^	0.05 [0.03, 0.09]

^(1)^ TKE values from 7 (00:05 UTC) to 28 (23:55 UTC) February 2020 and 16 (00:05 UTC) to 28 (11:15 UTC) June 2021 are available for summer and winter, respectively. ^(2)^ Indoor/outdoor air pollutant values from 23 June 2021 are not available. ^(3)^ Passive tube was exposed to the air from 21 to 28 June 2021.

## Data Availability

Data are available through the authors.

## Supplementary Materials

The following supporting information can be downloaded at https://www.mdpi.com/article/10.3390/ijerph20196823/s1, Figure S1: The local wind rose distributions at the ETSII site during the winter 2020 and summer 2021 campaigns. From top to bottom: all data, daytime and nighttime; Figure S2: Parameters measured indoors (In.) and outdoors (Out.) at the ETSII classroom during the winter campaign: PNC (Ultrafine Particle Number Concentration in cm^−3^); PM_10_, PM_2.5_ and PM_1_ (in μg·m^−3^); eBC (equivalent Black Carbon in μg·m^−3^); and NO and NO_2_ (in μg·m^−3^); Figure S3: Parameters measured indoors (In.) and outdoors (Out.) at the ETSII classroom during the summer campaign: PNC (Ultrafine Particle Number Concentration in cm^−3^); PM_10_, PM_2.5_ and PM_1_ (in μg·m^−3^); eBC (equivalent Black Carbon in μg·m^−3^); and NO and NO_2_ (in μg·m^−3^); Table S1: Air quality instruments installed at the ETSII classroom during the field campaigns; Table S2: Arithmetic mean and range (min–max) of one-hour data of pollutants (PNC, eBC, PM_10_, PM_2.5_, PM_1_, NO, NO_2_ and O_3_) measured at the university site during winter (6 February to 1 March 2020) and summer (14–23 June 2021) campaigns. UTC was used as the sampling time. All units are µg m-³ except PNC (Particle Number Concentration × 10³ in cm^−3^).
