# Supplementary material for: Real-Time Measurements of Indoor–Outdoor Exchange of Gaseous and Particulate Atmospheric Pollutants in an Urban Area
Source: Int J Environ Res Public Health. 2023 Sep 25;20(19):6823. doi: 10.3390/ijerph20196823 (PMC10572255; doi:10.3390/ijerph20196823)
Supplement: Supplementary file 1 [file ijerph-20-06823-s001.zip › ijerph-2515388-supplementary.pdf]

International Journal of
Environmental Research and Public Health

MDPI

Supplementary Materials

Real-Time Measurements of Indoor–Outdoor Exchange of Gaseous and Particulate Atmospheric Pollutants in an Urban Area

Elisabeth Alonso-Blanco [1,*], Francisco Javier Gómez-Moreno [1], Elías Díaz-Ramiro [1], Javier Fernández [1], Esther Coz [1], Carlos Yagüe [2], Carlos Román-Cascón [3], Adolfo Narros [4], Rafael Borge [4] and Begoña Artíñano [1]

1 Department of Environment, CIEMAT, 28040 Madrid, Spain
2 Department of Earth Physics and Astrophysics, Complutense University of Madrid, 28040 Madrid, Spain
3 Department of Applied Physics, Marine and Environmental Sciences Faculty, INMAR, CEIMAR, University of Cadiz, 11519 Puerto Real, Cádiz, Spain
4 Department of Chemical and Environmental Engineering, Technical University of Madrid (UPM), 28006 Madrid, Spain
* Correspondence: elisabeth.alonso@ciemat.es

Table S1. Air quality instruments installed at the ETSII classroom during the field campaigns.

Parameter	Instrument	Flow rate	Measurement size range	Air Sampling
O_3	Ozone Analyzer, Thermo Environmental Instruments model 49	2 L/min	0-500 ppb	Indoor[2]
O_3	Ozone Analyzer, Sabio model 6030	0.8 L/min	0-500 ppb	Outdoor[2]
NO, NO_2, NO_x	Thermo scientific 42i	0.7 L/min	0-500 ppb	Indoor[1]
NO, NO_2, NO_x	Thermo scientific 42i TL	0.3 L/min	0-500 ppb	Indoor[1]/Outdoor[2]
PM_{10}, $PM_{2.5}$, PM_1	Grimm model EDM365	1.2 L/min	Particles <10, <2.5 and <1 μm	Indoor
PM_{10}, $PM_{2.5}$, PM_1	Grimm model 11D	1.2 L/min	Particles <10, <2.5 and <1 μm	Outdoor
Particle number Concentration (PNC)	CPC, Aerosol Dynamics Inc. model MAGIC™	300 cm^{-3}/min	5 nm to 2.5 μm	Indoor
Particle number Concentration (PNC)	TSI 3772	1 L/min	Particles >10 nm	Outdoor
Equivalent Black Carbon (eBC)	MicroAeth® model MA200	100 ml/min	Particles <1.6 μm	Indoor
Equivalent Black Carbon (eBC)	MicroAeth® model AE51	50/100 ml/min	Particles <2.5 μm	Outdoor

[1] Winter 2020 campaign. [2] Summer 2021 campaign.

Table S2. Arithmetic mean and range [min-max] of one hour data of pollutants (PNC, eBC, PM_{10}, $PM_{2.5}$, PM_1, NO, NO_2 and O_3) measured at the university site during winter (6 February to 1 March 2020) and summer (14-23 June 2021) campaigns. UTC it was used in the sampling time. All units are $\mu g\ m^{-3}$ except PNC (particle number concentration × 10^3 in cm^{-3}).

		**PNC**	**PM_{10}**	**$PM_{2.5}$**	**PM_1**	**eBC**	**NO**	**NO_2**	**NO_2 Passive tubes**	**O_3**
	**Winter campaign**									
Indoor	All period	5.1 [1.0, 33.5]	4.4 [0.5, 35.8]	3.6 [0.4, 14.2]	3.3 [0.4, 12.5]	0.9 [0.1, 5.3]	38.3 [3.5, 126.4]	51.8 [24.4, 117.9]		-
Indoor	Week 1 (10 (11:05 UTC)–17 (13:05 UTC) February)	5.1 [2.1, 11.1]	5.4 [2.1, 30.9]	4.9 [2.1, 14.2]	4.7 [2, 11.9]	1.2 [0.4, 2.7]	32.1 [12.8, 79.4]	34.8 [29.8, 42.6]	34.1	-
Indoor	Week 2 (17 (13:06 UTC)–26 (13:22 UTC) February)	5.9 [1.0, 33.5]	3.0 [0.6, 35.8]	1.8 [0.6, 8.8]	1.5 [0.6, 7.1]	0.8 [0.1, 5.3]	54.6 [3.5, 126.4]	73.9 [25.8, 117.9]	39.0	-
Indoor	Stagnation episode days (19–25 February)	5.9 [1.5, 33.5]	3.0 [0.7, 35.8]	1.9 [0.7, 8.8]	1.6 [0.6, 7.1]	0.9 [0.2, 5.3]	64.2 [4.7, 126.4]	82.3 [32.2, 117.9]		-
Outdoor	All period	13.2 [1.7, 46.9]	30.3 [1.0, 241.8]	17.5 [0.7, 59.2]	-	1.6 [0.0, 8.5]	21.8 [1, 299.5]	46.7 [4.0, 152.3]		-
Outdoor	Week 1 (10 (11:05 UTC)–17 (13:05 UTC) February)	13.6 [3.1, 28.9]	31.1 [8.0, 73.8]	24.4 [5.3, 58.8]	-	2.2 [0.4, 7.0]	29.0 [1.2, 119]	47.5 [14.3, 80.7]	41.5	-
Outdoor	Week 2 (17 (13:06 UTC)–26 (13:22 UTC) February)	14.2 [1.7, 46.9]	19.5 [1.3, 66.2]	11.4 [1.5, 35.3]	-	1.4 [0.0, 8.5]	23.5 [1.0, 299.5]	52.0 [4.0, 152.3]	44.0	-
Outdoor	Stagnation episode days (19–25 February)	15.0 [3.3, 46.9]	21.2 [1.8, 66.2]	12.3 [1.5, 35.3]	-	1.6 [0.0, 8.5]	27.7 [1.0, 299.5]	58.0 [7.8, 152.3]		-
	**Summer campaign**									
Indoor	All period (1)	5.6 [2.0, 19.0]	2.4 [0.3, 8.7]	1.8 [0.3, 4.7]	1.4 [0.3, 3.0]	0.5 [0.2, 1.3]	7.0 [1.5, 16.4]	19.3 [11.3, 29.6]		9.2 [0.0, 66.6]
Indoor	Week 1 (14 (9:56 UTC)–21 (10:00 UTC) June)	5.2 [2.0, 14.8]	2.8 [0.6, 8.7]	2.1 [0.6, 4.7]	1.6 [0.6, 3.0]	0.5 [10.2, 1.3]	7.3 [2.0, 16.4]	20.0 [12.9, 29.6]	18.6	8.3 [0.4, 66.6]
Indoor	Week 2 (2 (10:01 UTC)–23 (11:22 UTC) June)	6.8 [2.0, 19.0]	1.4 [0.3, 7.8]	0.9 [0.3, 1.9]	0.6 [0.3, 1.2]	0.5 [0.2, 1.1]	5.4 [1.5, 14.1]	17.1 [11.3, 25.4]	16.2 (2)	12.2 [0.0, 64.3]
Outdoor	All period (1)	7.1 [2.2, 18.9]	9.0 [0.0, 40.4]	5.0 [0.0, 15.4]	2.5 [0.0, 6.7]	0.6 [0.1, 2.3]	3.1 [0.9, 14.9]	16.9 [5.8, 43.5]		86.2 [20.5, 177.4]
Outdoor	Week 1 (14 (9:56 UTC)–21 (10:00 UTC) June)	6.9 [2.2, 18.9]	11.0 [0.0, 40.4]	6.0 [0.0, 15.4]	2.9 [0.0, 6.7]	0.7 [0.2, 2.3]	3.3 [0.9, 14.9]	17.0 [5.8, 43.5]	15.1	90.8 [20.5, 177.4]
Outdoor	Week 2 (2 (10:01 UTC)–23 (11:22 UTC) June)	7.8 [2.9, 16.7]	3.6 [1.0, 9.0]	2.0 [0.6, 3.9]	1.2 [0.3, 2.4]	0.6 [0.1, 1.4]	2.8 [1.0, 7.7]	16.5 [6.0, 42.7]	14.5 (2)	72.1 [28.7, 113.1]

(1) Indoor/Outdoor air pollutant values from 23 June 2021 are not available. (2) Passive tube was exposed to the air from 21 to 28 June 2021.

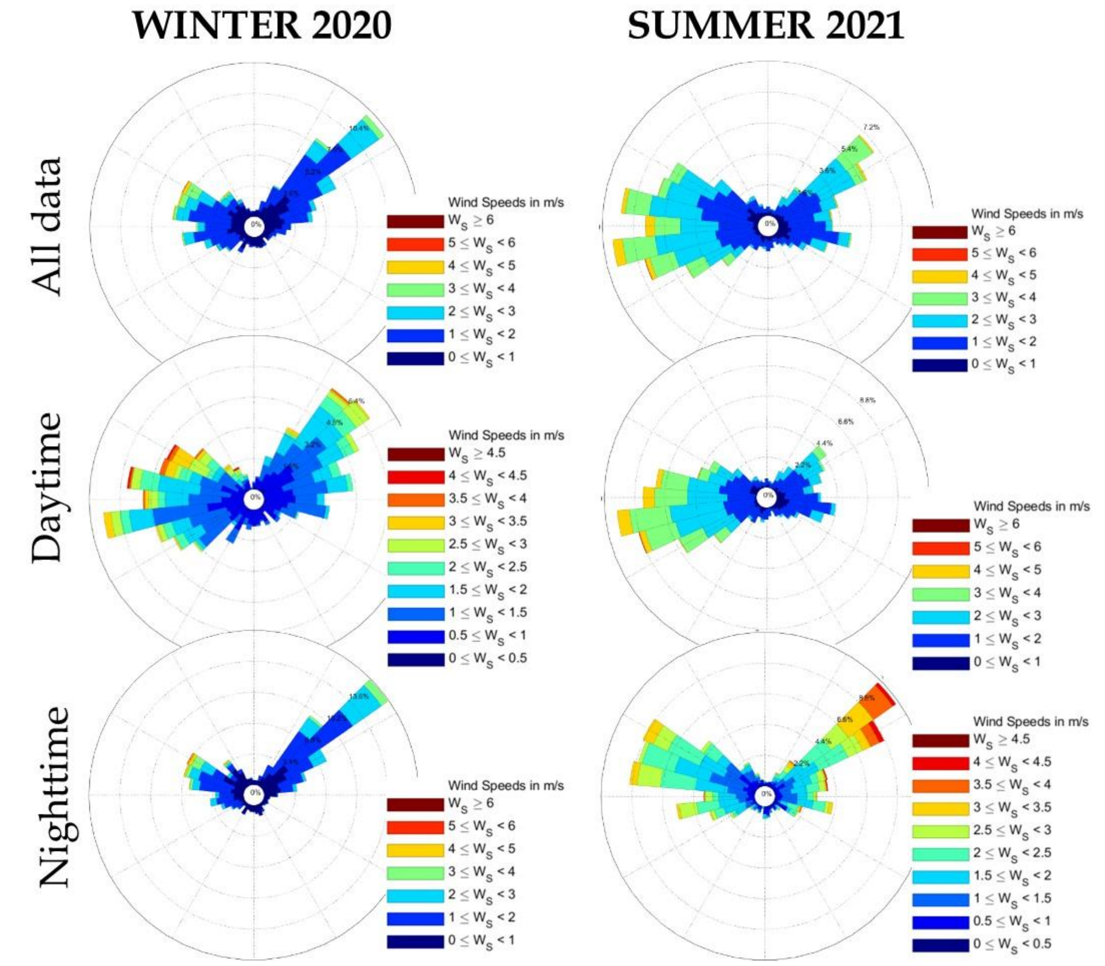

Figure S1. The local wind rose distributions at the ETSII site during the winter 2020 and summer 2021 campaigns. From top to bottom: all data, daytime and nighttime.

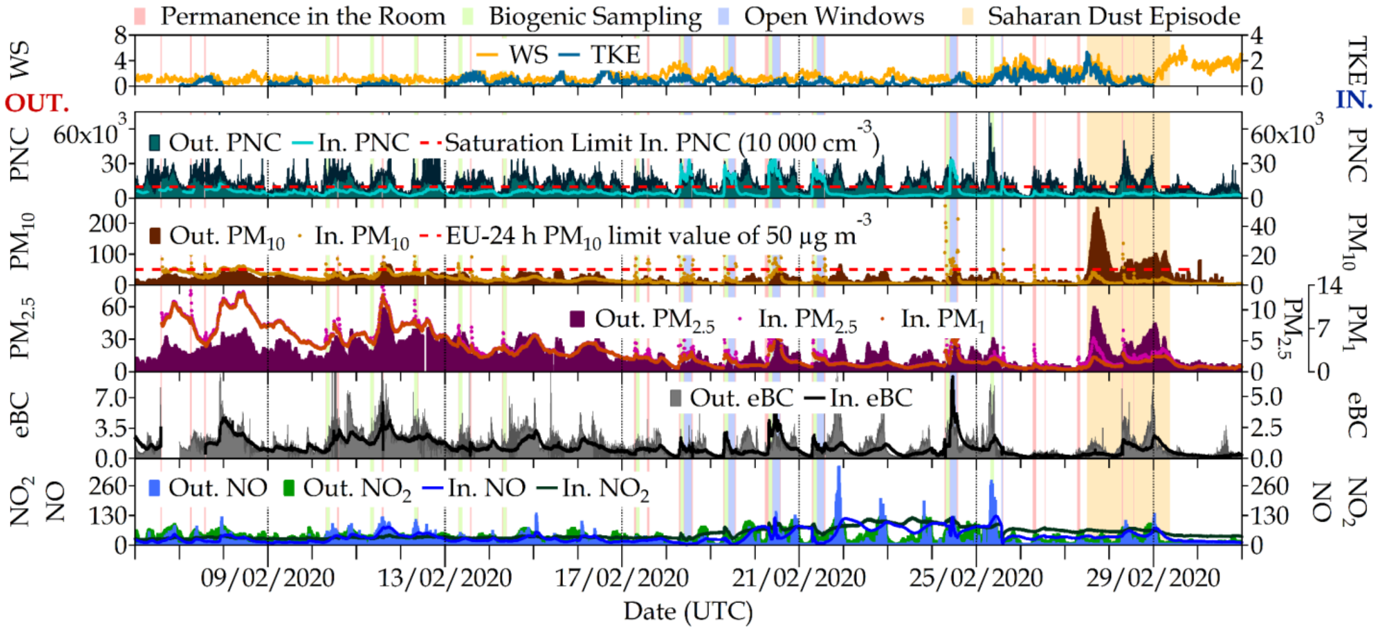

Figure S2. Parameters measured indoor (In.) and Outdoor (Out.) at the ETSII classroom during the winter campaign: PNC (Ultrafine Particle Number Concentration in cm^{-3}), PM_{10}, $PM_{2.5}$ and PM_1 (in µg·m^{-3}), eBC (equivalent Black Carbon in µg·m^{-3}) and NO and NO_2 (in µg·m^{-3}). WS (Wind Speed in m s^{-1}) and TKE (Turbulent Kinetic Energy in m^2 s^{-2}) values have been included as a proxy of ventilation conditions during the campaign. Outdoor measurements were represented by solid area whereas indoor measurements were represented by a line. For a better visualization, different scales have been used: Left vertical axis for Outdoor parameters and right vertical axis for Indoor parameters. Several events like Saharan dust, permanence in the classroom (instrument maintenance and data backup) or another type of sampling (biological sampling) and window opening are indicated in the figure.

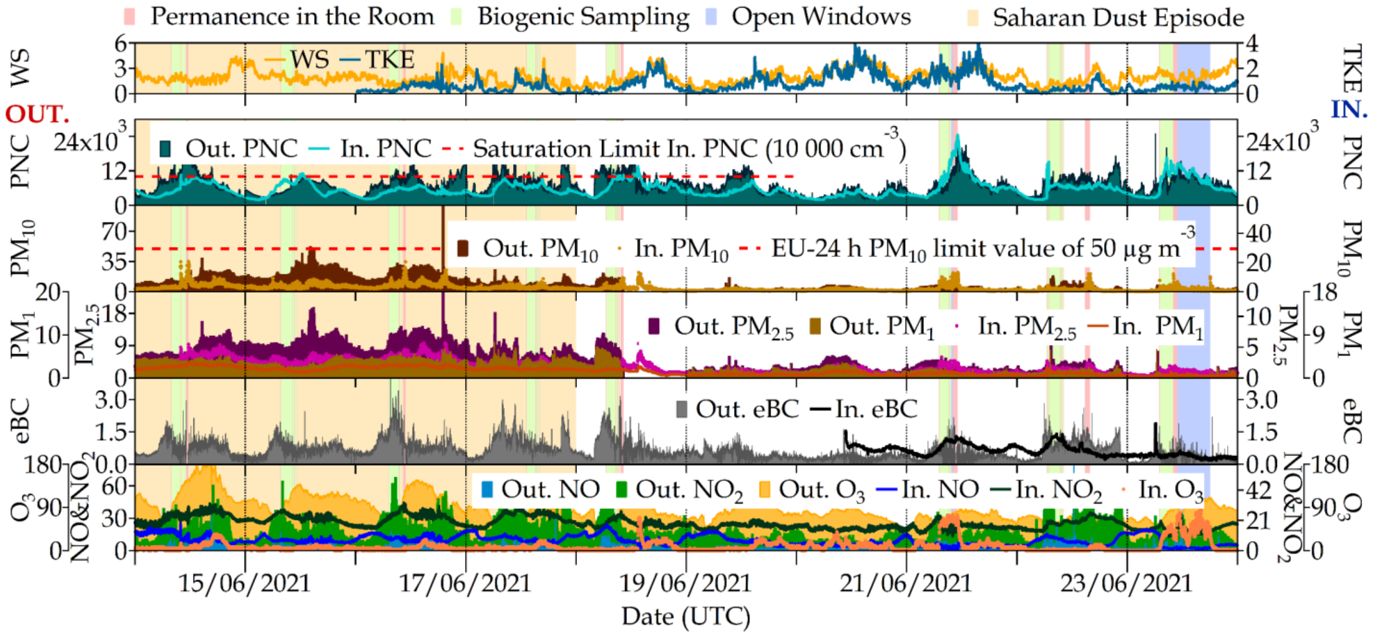

Figure S3. Parameters measured indoor (In.) and Outdoor (Out.) at the ETSII classroom during the summer campaign: PNC (Ultrafine Particle Number Concentration in cm^{-3}), PM_{10}, $PM_{2.5}$ and PM_1 (in $\mu g \cdot m^{-3}$), eBC (equivalent Black Carbon in $\mu g \cdot m^{-3}$) and NO and NO_2 (in $\mu g \cdot m^{-3}$). WS (Wind Speed in m s^{-1}) and TKE (Turbulent Kinetic Energy in m^2 s^{-2}) values have been included as a proxy of ventilation conditions during the campaign. Outdoor measurements were represented by solid area whereas indoor measurements were represented by a line. For a better visualization, different scales have been used: Left vertical axis for Outdoor parameters and right vertical axis for Indoor parameters. Several events like Saharan dust, permanence in the classroom (instrument maintenance and data backup) or another type of sampling (biological sampling) and window opening are indicated in the figure.
